# Multiple huge liver hydatid cysts in a child

**DOI:** 10.11604/pamj.2023.45.62.34567

**Published:** 2023-05-26

**Authors:** Takwa Mili, Yosra Ben Ahmed

**Affiliations:** 1 Department of Pediatric Surgery B, Children Hospital, Tunis, Tunisia

**Keywords:** Liver, hydatid cyst, child

## Image in medicine

A multiple cystic mass in the liver caused an 11-year-old girl who had a history of contact with animals to be referred to our unit. Abdominal ultrasound performed to investigate an abdominal mass revealed the disease. Physical examination revealed no icterus. There was a noticeable swelling in the right upper abdomen. The patient didn't exhibit any allergy symptoms like rhinitis or an urticarial rash. The liver had several low-density lesions, with the largest being a 12-cm-diameter unilocular hepatic cyst in segments IV, VII, and VIII. Two other unilocular cysts were detected in segments V and VI, which suggested liver hydatid cysts. Under general anesthesia, a syringe was used to aspirate intracystic fluid from the patient's largest unilocular hepatic cyst following liver exposure. The cyst was then opened, the internal capsule was taken out, and 15 minutes of hypertonic saline injection followed. Each cyst underwent a subtotal cystectomy with external draining of the remaining cavity. The residual cyst wall was examined intraoperatively for signs of bile leakage, and any apparent biliary holes were individually sutured in healthy tissue. Pathological analysis of the cyst tissue showed the presence of hydatid disease. The postoperative course was uncomplicated, and the patient had a good recovery following the procedure. Three months of ultrasonographic follow-up were carried out, and there was no sign of recurrence.

**Figure 1:**
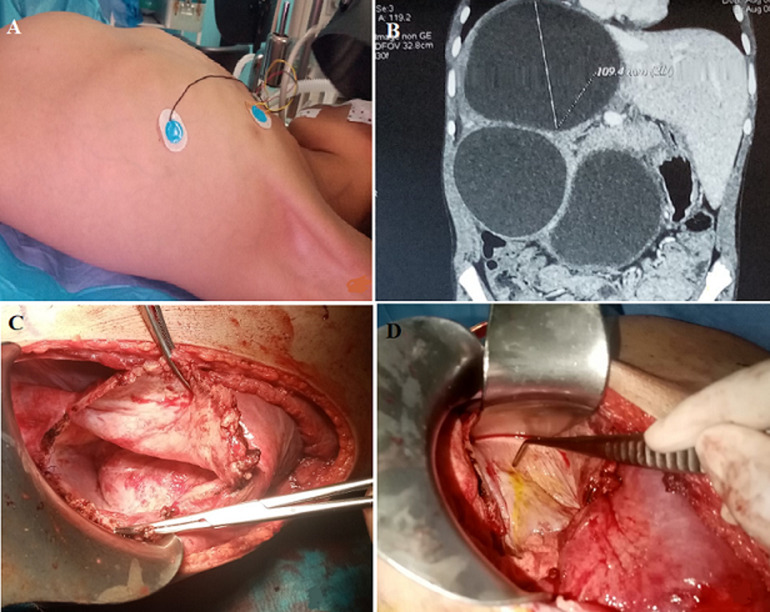
A) abdominal mass; B) abdominal computed tomography (CT) showing multiple huge liver hydatid cysts; C) residual cavity; D) cysto-biliary fistula

